# Exploring causal associations between multiple smoke exposures and neuroblastoma suppressor NBL1 via Mendelian randomization

**DOI:** 10.18332/tid/226573

**Published:** 2026-09-04

**Authors:** Siqi Xie, Shangeng Weng

**Affiliations:** 1 Department of Hepatopancreatobiliary Surgery, Fujian Abdominal Surgery Research Institute, The First Affiliated Hospital of Fujian Medical University, Fuzhou, Fujian, China; 2 Department of Hepatopancreatobiliary Surgery, National Regional Medical Center, Binhai Campus of the First Affiliated Hospital, Fujian Medical University, Fuzhou, Fujian, China; 3 Fujian Children’s Hospital (Fujian Branch of Shanghai Children’s Medical Center), College of Clinical Medicine for Obstetrics and Gynecology and Pediatrics, Fujian Medical University, Fuzhou, Fujian, China

**Keywords:** tobacco exposure, neuroblastoma, NBL1, Mendelian randomization

## Abstract

**INTRODUCTION:**

Neuroblastoma suppressor of tumorigenicity 1 (NBL1) is an established tumor suppressor and prognostic biomarker in neuroblastoma (NB). Although tobacco exposure is a well-known carcinogenic factor, its potential effect on NBL1 expression remains unclear. This two-sample Mendelian randomization (MR) study aimed to explore potential links between diverse smoking exposures, circulating NBL1 levels and NB risk.

**METHODS:**

This study performed a two-sample MR analysis to investigate the causal effects of maternal smoking around birth (66 single-nucleotide polymorphisms, SNPs), household smoking exposure (17 SNPs), and smoking initiation (37 SNPs) on circulating NBL1 levels using large-scale GWAS summary statistics. Causal estimates were obtained using IVW, weighted median, and MR-Egger methods, with sensitivity, reverse MR, and validation analyses conducted to assess robustness and directionality.

**RESULTS:**

No evidence of a causal association was observed between maternal smoking around birth or household smoking exposure and NBL1 levels. In contrast, genetically predicted smoking initiation was associated with lower NBL1 expression (IVW: OR=0.70; 95% CI: 0.50–0.99, p=0.04). The weighted median method failed to reach statistical significance (OR=0.642; 95% CI: 0.40–1.04, p=0.07), whereas MR-Egger regression yielded a non-significant estimate (OR=0.39; 95% CI: 0.06–2.56, p=0.34). Sensitivity analyses indicated no substantial pleiotropy or influential outliers. Reverse MR analysis provided no evidence that NBL1 influences smoking behavior. Additional validation analyses showed no causal association between smoking initiation and NB risk.

**CONCLUSIONS:**

Prenatal and household smoking exposures do not appear to affect NBL1 expression. Genetically predicted smoking initiation was associated with reduced NBL1 expression, but not with neuroblastoma risk.

## INTRODUCTION

Tobacco smoking remains one of the leading preventable causes of cancer worldwide. Cigarette smoke contains more than 7000 chemical compounds, including at least 69 recognized carcinogens such as polycyclic aromatic hydrocarbons and tobacco-specific nitrosamines^1,2^. The International Agency for Research on Cancer (IARC) has classified tobacco smoking as a Group 1 carcinogen and has established causal associations with at least 17 cancer types, including cancers of the lung, esophagus, bladder, liver, pancreas, and cervix^3^. Smoking-related carcinogenesis is mediated through multiple mechanisms, including DNA damage, somatic mutations in oncogenes and tumor suppressor genes, chronic inflammation, oxidative stress, and epigenetic dysregulation^4^. In addition to active smoking, secondhand smoke exposure is also associated with increased cancer risk among non-smokers^1^.

Emerging evidence further suggests that maternal smoking during pregnancy may exert long-term health effects on offspring, including an increased risk of certain malignancies later in life^5^. Neuroblastoma (NB) is the most common extracranial solid malignancy of childhood, accounting for approximately 7% of pediatric cancers and 15% of childhood cancer-related deaths worldwide^6^. Despite substantial advances in multimodal treatment strategies, outcomes remain poor for patients with high-risk disease, largely owing to metastatic progression and treatment resistance^7^. Unlike most adult cancers, NB is characterized by a relatively low mutational burden but frequent chromosomal abnormalities, including deletions of chromosome arms 1p and 11q and gain of 17q^8^. These genomic alterations highlight the importance of tumor suppressor genes in NB pathogenesis. Neuroblastoma suppressor of tumorigenicity 1 (NBL1), also known as DAN, is a secreted glycoprotein encoded by a gene located on chromosome 1p36.12, a region commonly deleted in NB^9^. NBL1 functions as an antagonist of bone morphogenetic proteins (BMPs) and negatively regulates BMP-Smad signaling, thereby inhibiting cell proliferation and promoting cellular differentiation^10^. Tobacco carcinogens drive epigenetic silencing and chromosomal damage at the NBL1 gene locus, reducing NBL1 protein abundance and disrupting tumor-suppressive BMP signal^11^. Downregulation or silencing of NBL1 is commonly observed in NB tissues, especially in high-risk cases with MYCN amplification. Genomic deletion of 1p covering the NBL1 locus further leads to its reduced expression, which is closely correlated with advanced clinical stage, aggressive tumor phenotypes, and poor patient survival^12^. Functional experiments have verified that ectopic NBL1 expression suppresses the malignant behaviors of NB cells. Collectively, NBL1 acts as a vital tumor suppressor and independent prognostic biomarker in neuroblastoma^13^.

Mendelian randomization (MR) is an analytical approach that uses genetic variants as instrumental variables to infer causal relationships between exposures and outcomes. Because genetic variants are randomly allocated at conception, MR analyses are less vulnerable to residual confounding and reverse causality than traditional observational studies. The increasing availability of large-scale genome-wide association studies (GWAS) has further enhanced the utility of MR for investigating potential causal mechanisms in complex diseases^14^. Two-sample MR, in particular, utilizes summary-level GWAS data from separate but comparable populations to estimate the causal effect of an exposure on an outcome.

Existing observational links between tobacco smoke and childhood neuroblastoma are limited by confounding, unknown NBL1-mediated mechanisms, and incomplete smoking phenotype stratification, with no two-sample MR evidence clarifying the full causal pathway connecting diverse smoking exposures, circulating NBL1, and neuroblastoma risk. To our knowledge, this is the first study to investigate the potential causal relationship between smoking-related exposures and NBL1 using an MR framework. Two-sample MR analyses were implemented to quantify causal effects of three smoking phenotypes (perinatal smoking, household smoke exposure, personal smoking initiation; GWAS IDs: ukb-b-17685, ukb-a-18, ieu-b-4877) on circulating NBL1 concentrations (prot-a-2003).

## METHODS

### Data source

This study was conducted in accordance with the STROBE-MR reporting guidelines and the core assumptions of Mendelian randomization (MR) (Supplementary file Table S1)^15^.

This is a bidirectional two-sample Mendelian randomization study design using genome-wide association study (GWAS) summary statistics for potential inference. Summary-level GWAS data for maternal smoking around birth were obtained from the UK Biobank dataset (ukb-b-17685), comprising 397732 participants and 9851867 single-nucleotide polymorphisms (SNPs)^16^. Data for household smoking exposure were derived from the UK Biobank dataset (ukb-a-18), which included 311142 individuals of European ancestry and 10894596 SNPs^17^. GWAS summary statistics for smoking initiation were obtained from the IEU OpenGWAS database (ieu-b-4877), including 311629 cases, 321173 controls, and 11802365 SNPs^18^.

Outcome data for neuroblastoma suppressor of tumorigenicity 1 (NBL1) were obtained from the Genomic Atlas of the Human Plasma Proteome (prot-a-2003). In this study, 3622 plasma proteins were quantified using the SomaScan 4.0 platform in 3301 healthy European participants from the INTERVAL cohort^19^. Detailed characteristics of all datasets are summarized in Table 1.

**Table 1:** Characteristics of the datasets

*Exposure*	*Web source*	*Sample size*	*SNP size*	*First author*	*Consortium*	*Year*	*Population*
Maternal smoking around birth	UK Biobank (ukb-b-17685)	397732	9851867	B. Elsworth	MRC-IEU	2018	European
Smoking in household	UK Biobank (ukb-a-18)	311142	10894596	Neale	Neale Lab	2017	European
Smoking initiation	IEU GWAS database (ieu-b-4877)	311629	11802365	Liu	GSCAN	2019	European
**Outcome**							
Neuroblastoma suppressor of tumorigenicity 1 (NBL1)	Genomic atlas of the human plasma proteome (prot-a-2003) (2018, Nature)	3301	10534735	B.B. Sun	NA	2018	European

### Instrumental variable selection

Instrumental variables (IVs) were selected using stringent criteria to ensure the validity of causal inference. SNPs associated with each exposure at genome-wide significance (p<5×10^-8^) were selected. For household smoking exposure, the threshold was relaxed to p<5×10^-6^ because of the limited number of eligible variants. To ensure independence among IVs, linkage disequilibrium (LD) clumping was performed using an r^2^ threshold of <0.001 within a 10000kb window. SNPs with a minor allele frequency below 1% were excluded. Potentially pleiotropic variants associated with known confounders, cancer-related traits, or lung-development phenotypes were identified and removed using the *FastTraitR* package (version 1.0.1).

The proportion of variance explained (R^2^) by each SNP was calculated as:

R^2^ = [2β^2^×EAF×(1-EAF)]/[2β^2^×EAF×(1-EAF) + 2SE^2^×N×EAF×(1-EAF)]

where β represents the SNP–exposure effect estimate, EAF denotes the effect allele frequency, SE is the standard error, and N is the sample size. Instrument strength was evaluated using the F statistic, with F >10 considered indicative of sufficient instrument strength.

### Statistical analysis

Two-sample MR analyses were performed using R software (version 4.3.2) and the *TwoSampleMR* package (version 0.6.29). The inverse-variance weighted (IVW) method^20,21^ was used as the primary analytical approach. Weighted median^22^ and MR-Egger regression analyses^23^ were performed as complementary methods to evaluate the robustness of the findings. Causal effects are reported as odds ratios (ORs) with corresponding 95% confidence intervals (CIs). A p<0.05 was considered statistically significant.

### Sensitivity analyses

Several sensitivity analyses were conducted to assess the validity of the MR assumptions: 1) MR-Egger intercept test to detect horizontal pleiotropy; 2) Cochran’s Q test to evaluate heterogeneity among instrumental variables; and 3) Leave-one-out analysis to determine whether individual SNPs disproportionately influenced the overall estimates^24^.

## RESULTS

### Associations between smoking exposures and neuroblastoma suppressor of tumorigenicity 1 (NBL1)

The causal effects of three smoking-related exposures on circulating NBL1 levels are summarized in Table 2. For maternal smoking around birth, 66 independent SNPs were selected as instrumental variables at the genome-wide significance threshold (p<5×10^-8^). No evidence of a causal association with NBL1 levels was observed using the IVW method (OR=0.40; 95% CI: 0.12–1.30, p=0.13). Consistent null results were obtained using the weighted median (OR=0.55, 95% CI: 0.11–2.86, p=0.48) and MR-Egger methods (OR=0.08; 95% CI: 0.00–3.59, p=0.12).

**Table 2:** Mendelian randomization results for the association between the three exposures and NBL1, IEU OpenGWAS 2017, 2018, 2019 and 2023

*Exposure*	*Outcome (N=3301)*	*Forward MR*	*β*	*SE*	*OR (95% CI)*	*p*
Maternal smoking around birth (N=397732)	NBL1	MR Egger	-2.51	1.93	0.08 (0.00–3.59)	0.20
		Weighted median	-0.60	0.84	0.55 (0.11–2.86)	0.48
		IVW	-0.92	0.60	0.40 (0.12–1.30)	0.13
		Simple mode	-1.69	2.01	0.19 (0.00–9.54)	0.41
		Weighted mode	-1.37	1.81	0.26 (0.01-8.91)	0.45
Smoking in household (N=311142)	NBL1	MR Egger	0.52	5.39	1.68 (0.00–64768.32)	0.92
		Weighted median	0.35	2.22	1.42 (0.018–110.176)	0.88
		IVW	1.31	1.95	3.72 (0.08–171.13)	0.50
		Simple mode	-1.91	3.98	0.15 (0.00–357.88)	0.64
		Weighted mode	-1.70	3.94	0.18 (0.00–417.18)	0.67
Smoking initiation (N=311629)	NBL1	MR Egger	-0.93	0.96	0.39 (0.06–2.56)	0.34
		Weighted median	-0.44	0.24	0.64 (0.40–1.04)	0.07
		IVW	-0.35	0.17	0.70 (0.50–0.99)	0.04
		Simple mode	-0.52	0.50	0.60 (0.23–1.57)	0.30
		Weighted mode	-0.58	0.45	0.56 (0.23–1.34)	0.20

NBL1: neuroblastoma suppressor of tumorigenicity 1. IVW: inverse variance weighted. OR: odds ratio. CI: confidence interval. SE: standard error.

For household smoking exposure, 17 independent SNPs were included following instrument selection at p<5×10^-6^. No significant association with NBL1 levels was identified by the IVW method (OR=3.72; 95% CI: 0.08–171.13, p=0.50), weighted median estimator (OR=1.42; 95% CI: 0.02–110.18, p=0.88), or MR-Egger regression (OR=1.68; 95% CI: 0.00–64768.32, p=0.92).

**Figure 1:**
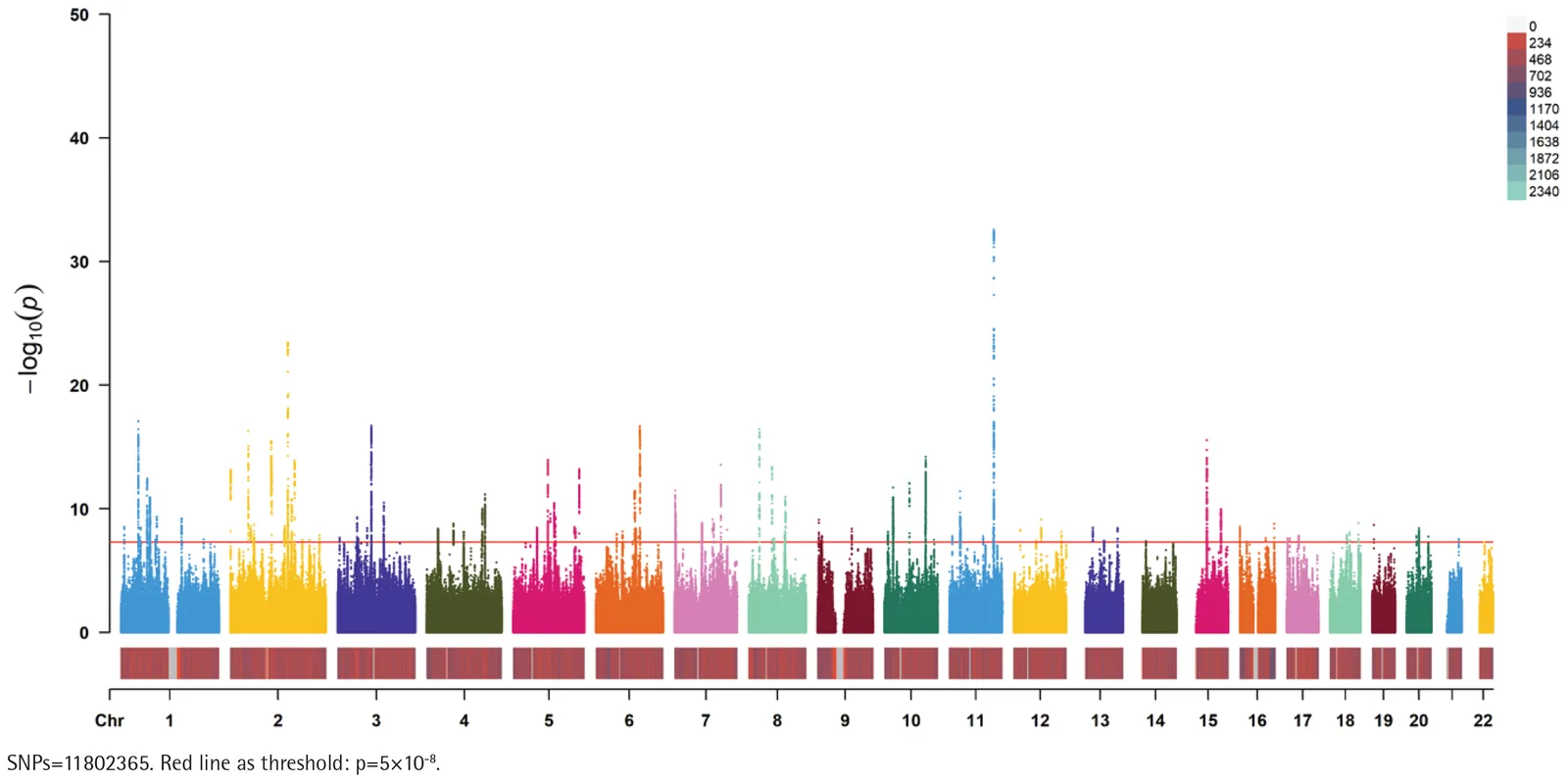
Manhattan plot showing genome-wide significant SNPs associated with smoking initiation, data from IEU OpenGWAS in 2019

**Figure 2:**
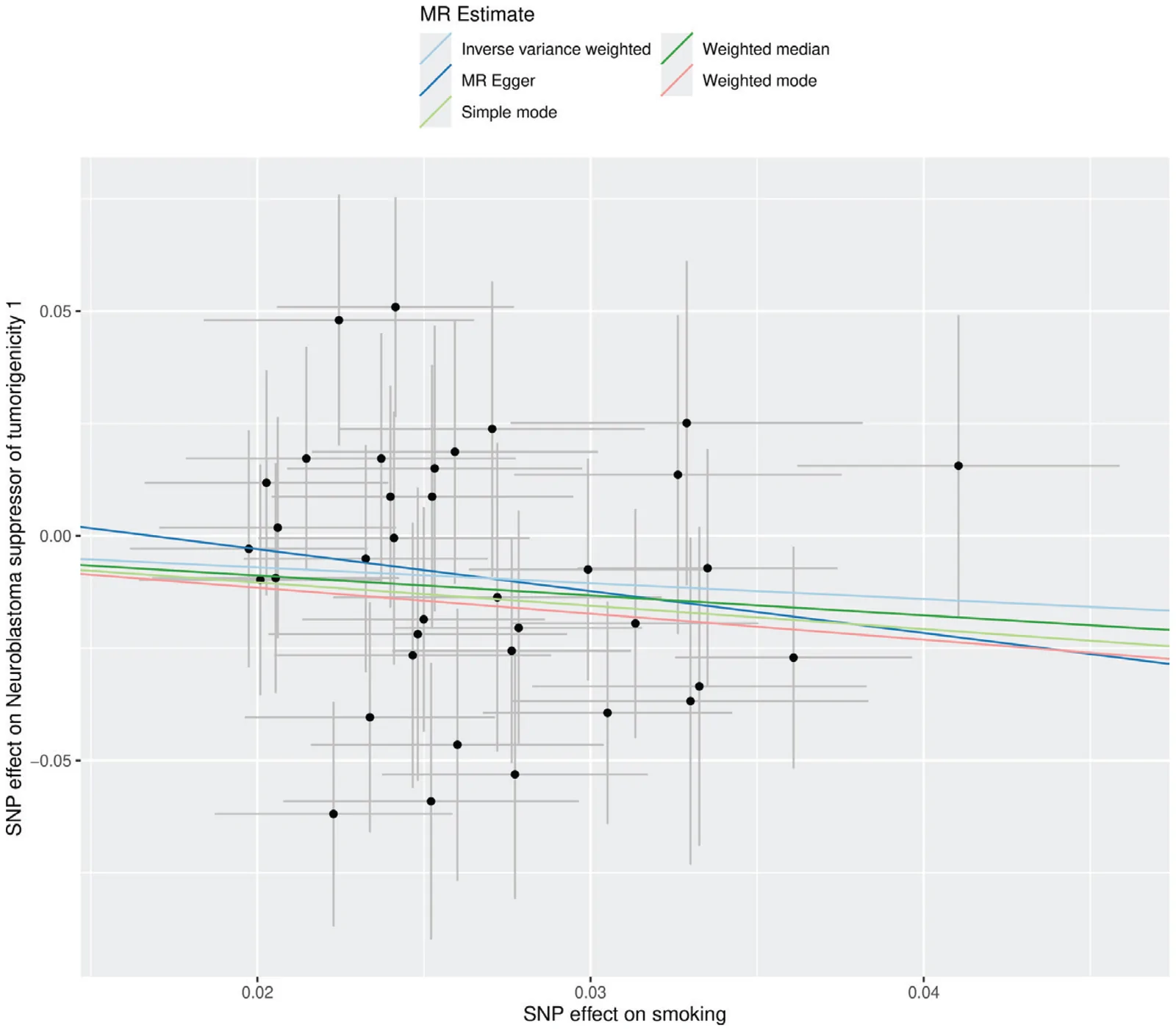
Scatter plot showing the associations of instrumental SNPs with smoking initiation and NBL1 expression. Each point represents an SNP effect estimate with 95% confidence intervals. The fitted lines correspond to causal estimates obtained using the IVW, weighted median, and MR-Egger methods, IEU OpenGWAS 2018 and 2019 (N=314930)

**Figure 3:**
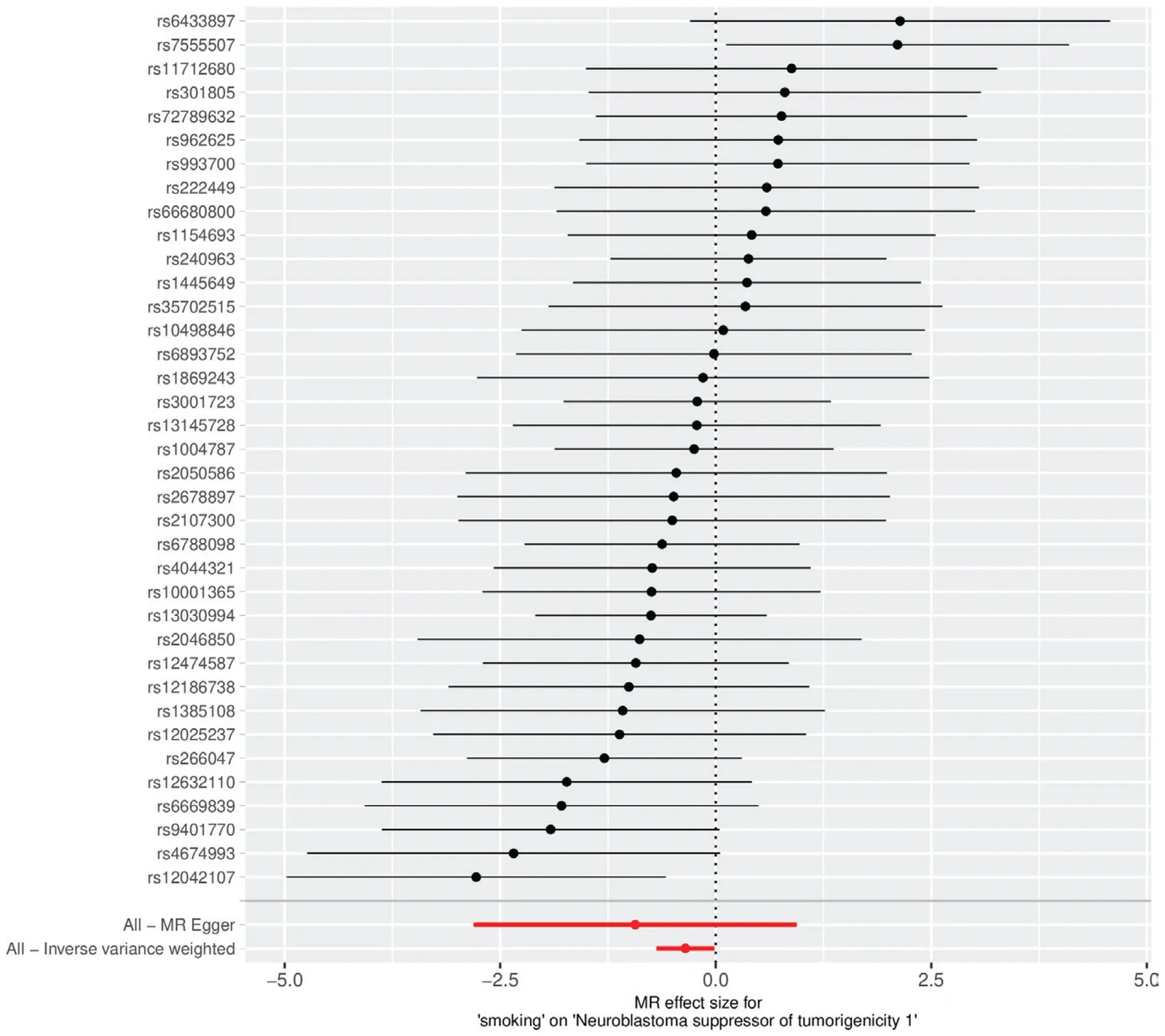
Forest plot of SNP-specific Mendelian randomization estimates for the association between smoking initiation and NBL1 expression. Black dots indicate effect estimates, and horizontal bars represent 95% confidence intervals, data from 2018 and 2019 (N=311629 for smoking initiation; N=3301 for NBL1)

For smoking initiation, 37 genome-wide significant SNPs (p<5×10^-8^) were selected as instrumental variables following stringent quality control and clumping procedures (Figure 1). The primary IVW analysis demonstrated a significant inverse association between genetically predicted smoking initiation and circulating NBL1 levels (OR=0.70; 95% CI: 0.50–0.99, p=0.04). The weighted median analysis yielded a similar effect estimate, although statistical significance was not reached (OR=0.64; 95% CI: 0.40–1.04, p=0.07). MR-Egger regression showed a consistent direction of effect but with wider confidence intervals (OR=0.39; 95% CI: 0.06–2.56, p=0.34). Scatter plots (Figure 2) and forest plots (Figure 3) demonstrated overall concordance across MR approaches.

### Detailed information of the included SNPs

Table 3 provides comprehensive details for each SNP, including the effect allele (EA) and its frequency (EAF) in the exposure, which is significantly associated with the outcome. It also includes estimates of their associations with smoking and NBL1, encompassing β values, standard errors (SE), and corresponding p values.

**Table 3:** Association analysis for smoking-decreasing GWAS risk alleles with the NBL1, IEU OpenGWAS 2017, 2018, 2019 and 2023

*CHR*	*Position*	*SNPs*	*EA*	*EAF*	*Smoking*			*NBL1*		
					*β*	*SE*	*p*	*β*	*SE*	*p*
1	154205120	rs12025237	C	0.12	-0.03	0.01	6.39E-10	0.04	0.04	0.31
1	91196176	rs12042107	C	0.53	-0.02	0.00	4.24E-10	0.06	0.03	0.01
1	210304319	rs2046850	T	0.19	-0.02	0.00	3.01E-08	0.02	0.03	0.50
1	87905828	rs2050586	C	0.36	-0.02	0.00	3.01E-08	0.01	0.03	0.71
1	44037685	rs3001723	A	0.32	0.03	0.00	8.22E-18	-0.01	0.03	0.78
1	8481016	rs301805	G	0.56	0.02	0.00	2.83E-09	0.02	0.02	0.49
1	50625979	rs6669839	T	0.20	0.03	0.00	3.30E-09	-0.05	0.03	0.12
1	73766037	rs7555507	T	0.50	-0.02	0.00	1.12E-11	-0.05	0.02	0.04
2	45159091	rs1004787	A	0.58	0.03	0.00	5.36E-17	-0.01	0.02	0.76
2	162802993	rs12474587	T	0.40	0.03	0.00	1.22E-14	-0.03	0.03	0.30
2	146143090	rs13030994	A	0.49	0.04	0.00	3.35E-24	-0.03	0.02	0.28
2	155682556	rs1445649	C	0.53	0.02	0.00	1.69E-11	0.01	0.02	0.72
2	200937901	rs2107300	G	0.85	-0.03	0.00	3.34E-08	0.01	0.03	0.69
2	104088751	rs266047	A	0.53	-0.03	0.00	3.33E-16	0.04	0.02	0.11
2	58169418	rs2678897	A	0.63	0.02	0.00	3.52E-08	-0.01	0.03	0.71
2	137542847	rs35702515	T	0.16	0.03	0.00	2.42E-09	0.01	0.03	0.78
2	226332033	rs4674993	G	0.21	-0.03	0.00	1.32E-08	0.06	0.03	0.05
2	182034448	rs6433897	C	0.75	0.02	0.00	3.17E-08	0.05	0.03	0.09
3	117804154	rs1154693	G	0.86	0.03	0.00	3.12E-11	0.01	0.04	0.71
3	75009019	rs11712680	C	0.17	-0.03	0.00	3.47E-09	-0.02	0.03	0.47
3	50224225	rs12632110	G	0.65	-0.02	0.00	4.70E-10	0.04	0.03	0.11
3	5724536	rs1869243	C	0.48	0.02	0.00	3.01E-08	0.00	0.03	0.91
3	85985324	rs66680800	T	0.40	-0.02	0.00	2.86E-08	-0.01	0.03	0.65
3	85624131	rs6788098	T	0.62	-0.03	0.00	1.94E-17	0.02	0.03	0.45
4	147797214	rs10001365	A	0.41	-0.02	0.00	6.74E-12	0.02	0.03	0.46
4	140927812	rs13145728	C	0.36	-0.02	0.00	2.18E-10	0.01	0.03	0.83
4	28473524	rs962625	G	0.24	0.02	0.00	4.26E-09	0.02	0.03	0.54
4	67825894	rs993700	C	0.77	-0.03	0.00	1.53E-09	-0.02	0.03	0.52
5	103816655	rs12186738	T	0.15	-0.03	0.01	3.46E-11	0.03	0.04	0.35
5	154839646	rs1385108	T	0.24	0.02	0.00	2.98E-09	-0.03	0.03	0.36
5	166989513	rs4044321	G	0.64	-0.03	0.00	6.22E-14	0.02	0.03	0.44
5	60374912	rs6893752	G	0.77	-0.02	0.00	3.30E-09	0.00	0.03	0.98
5	106834363	rs72789632	T	0.12	-0.03	0.01	4.94E-10	-0.03	0.04	0.49
6	67405337	rs10498846	T	0.47	0.02	0.00	6.77E-09	0.00	0.02	0.93
6	52916062	rs222449	T	0.79	-0.03	0.00	1.08E-08	-0.02	0.03	0.63
6	111644332	rs240963	C	0.84	-0.04	0.00	2.15E-17	-0.02	0.03	0.65
6	98748008	rs9401770	A	0.27	0.03	0.00	3.48E-12	-0.05	0.03	0.06

CHR: chromosome. EA: effect allele. EAF: effect allele frequency. SE: standard error. SNPs: single-nucleotide polymorphisms. NBL1: neuroblastoma suppressor of tumorigenicity 1. N=397732 for maternal smoking around birth. N=311142 for smoking in household. N=311629 for smoking initiation. N=3301 for neuroblastoma suppressor of tumorigenicity 1.

### Sensitivity analysis

**Figure 4:**
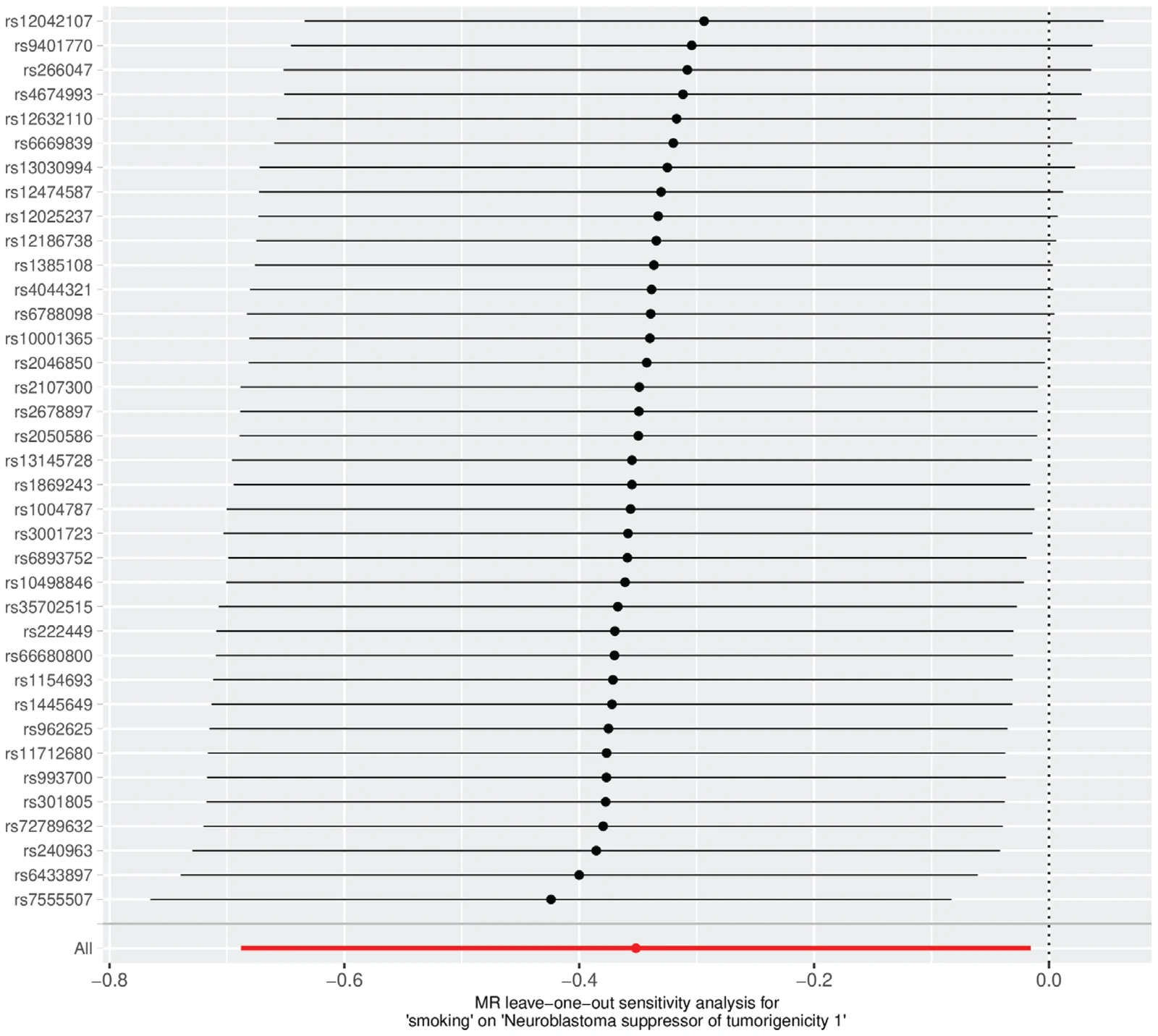
Leave-one-out analysis assessing the influence of individual SNPs on the estimated effect of smoking initiation on NBL1 expression, IEU OpenGWAS 2018 and 2019 (N=314930)

No evidence of directional pleiotropy was detected for any exposure. Cochran’s Q test indicated no significant heterogeneity for maternal smoking around birth or smoking initiation, whereas heterogeneity was observed for household smoking exposure (Table 4). Leave-one-out analyses showed that no individual SNP disproportionately influenced the observed association between smoking initiation and NBL1 expression (Figure 4).

**Table 4:** Heterogeneity and pleiotropy tests, IEU OpenGWAS 2017, 2018, 2019 and 2023

*Exposure*	*Outcome*	*Heterogeneity test*				*Pleiotropy test*
		*IVW Q*	*p*	*MR-Egger Q*	*p*	*MR-Egger p*
ukb-b-17685	prot-a-2003	48.81	0.93	48.06	0.93	039
ukb-a-18		28.04	0.03	27.99	0.02	0.88
ieu-b-4877		35.06	0.51	34.68	0.48	0.54

IVW: inverse variance weighted. MR: Mendelian randomization. A p<0.05 was considered statistically significant. N=397732 for maternal smoking around birth. N=311142 for smoking in household. N=311629 for smoking initiation. N=3301 for neuroblastoma suppressor of tumorigenicity 1.

### The reverse MR

To assess potential reverse causality, reverse MR analyses were performed using NBL1-associated SNPs as instrumental variables. Because no variants reached genome-wide significance (p<5×10^-8^), 15 independent SNPs were selected using a relaxed threshold of p<5×10^-6^. The IVW analysis provided no evidence that NBL1 levels causally influence smoking initiation (Supplementary file Table S2). Additionally, leave-one-out sensitivity analysis revealed heterogeneity across SNPs when assessed individually against the collective set (Supplementary file Figure 1).

### Validation analysis using neuroblastoma risk

To verify that the observed inverse association was specific to NBL1 rather than NB, we conducted an additional MR analysis of smoking and NB using the ieu-a-816 GWAS dataset^25^. The dataset comprised 1627 NB cases, 3254 controls, and 468788 SNPs, from which 10 instrumental SNPs were selected. No causal association between smoking and NB was identified across any MR method (IVW: OR=1.05; 95% CI: 0.36–3.05, p=0.93; weighted median: OR=0.93; 95% CI: 0.21–4.04, p=0.92; MR-Egger: OR=0.09; 95% CI: 0.00–3.79, p=0.24) (Supplementary file Table S3).

## DISCUSSION

To ensure robust inference, this study selected maternal smoking around birth, smoking initiation, and household smoking exposure as instrumental variables (IVs), representing intergenerational, individual behavioral, and secondhand smoking effects, respectively. Potentially pleiotropic variants linked to known confounders, including cancer-related dyslipidemia (‘rs6728726’, ‘rs2378662’) and lung development traits (‘rs3800227’)^26^, were excluded using the *FastTraitR* package. All IVs exhibited sufficient strength (F-statistic >10). Using complementary MR approaches, including inverse-variance weighted (IVW), weighted median, and MR-Egger analyses, this study identified a potential inverse association between genetically predicted smoking initiation and NBL1 expression. In contrast, maternal smoking around birth and household smoking exposure showed no significant effects on NBL1. Sensitivity analyses detected no evidence of horizontal pleiotropy or influential outliers, and replication across independent GWAS datasets further strengthened the robustness of the findings. Reverse MR analyses provided no evidence that NBL1 expression influences smoking behavior, supporting the inferred causal direction.

Tobacco control remains a major global public health priority. Secondhand smoke exposure continues to represent a substantial environmental risk, disproportionately affecting vulnerable populations such as children, pregnant women, and non-smokers^1^. Cigarette smoke contains thousands of chemical compounds, including nicotine, tar, and formaldehyde. These toxic components induce oxidative stress and trigger the release of pro-inflammatory cytokines, including IL-6, IL-8, IL-17, and TNF-α, thereby amplifying systemic inflammation and promoting carcinogenesis^27^.

NBL1 (known as DAN) is a well-characterized antagonist of bone morphogenetic protein (BMP) signaling, particularly BMP-2 and BMP-7. It has been widely studied in developmental and tumor biology as a key modulator of cellular differentiation and proliferation^28^. Emerging evidence also suggests that NBL1 may attenuate neuroinflammation through BMP-dependent microglial activation pathways^29^. In pulmonary vascular disease models, NBL1 has been shown to suppress PDGF-BB-induced proliferation of human pulmonary artery smooth muscle cells, thereby inhibiting vascular remodeling^26^. Beyond neuroblastoma, NBL1 also exerts tumor-suppressive effects in several malignancies, including prostate cancer and small-cell lung cancer^30,31^.

Despite growing evidence linking smoking to multiple malignancies^11^, its potential role in NB and the underlying molecular mechanisms remain largely unexplored. The study performed a comprehensive bidirectional MR analysis to investigate the causal relationships between smoking-related exposures, NBL1 expression, and NB risk. Compared with conventional observational studies, MR analyses reduce susceptibility to residual confounding and reverse causation. These findings have several important implications. First, maternal smoking exposure does not appear to increase offspring NB risk through NBL1-mediated mechanisms. Second, secondhand smoke exposure is unlikely to contribute to NB development via alterations in NBL1 expression. Third, although smoking initiation was associated with reduced NBL1 expression, this relationship is unlikely to influence NB because smoking exposure typically occurs after the peak incidence period of this pediatric malignancy. Instead, smoking-induced NBL1 downregulation may be more relevant to the pathogenesis of adult-onset cancers, a hypothesis further supported by these supplementary validation analyses.

### Limitations

Several limitations should be acknowledged. First, all GWAS datasets were derived from populations of European ancestry, which may limit the generalizability of these findings. Second, although no substantial horizontal pleiotropy was detected, residual pleiotropic effects cannot be completely excluded. Third, heterogeneity was observed among some instrumental variables, particularly in analyses involving household smoking and NBL1; however, this did not materially alter the causal estimates. Fourth, the two-sample MR design remains vulnerable to potential violations of core MR assumptions and cannot account for unmeasured gene–environment interactions. Finally, because relatively few SNPs were available for certain exposures, a relaxed significance threshold (p<5×10^-6^) was adopted. Nevertheless, stringent linkage disequilibrium filtering (r^2^<0.001 within 10000 kb) and F-statistic exceeding 10 minimized the likelihood of weak-instrument bias. Moreover, the observed effect size was modest, indicating that the clinical relevance of smoking-related NBL1 alterations may be limited despite their potential biological significance. Finally, parallel testing of three smoking exposures, bidirectional MR designs, and neuroblastoma validation could increase type I error risk, meaning the nominally significant association between smoking initiation and NBL1 needs external replication to avoid false positives. In addition, accessible GWAS instruments merely define binary smoking initiation, lacking genetic markers for smoking intensity, pack-years, and duration, which prevents dose–response assessment.

### Future research

To resolve these limitations and outstanding research questions, future investigations are warranted, encompassing cross-ancestry MR replication, *in vitro* functional assays validating smoke-induced transcriptional repression of NBL1, MR analyses evaluating dose-dependent smoking effects, prospective pediatric cohort studies monitoring smoke exposure and circulating NBL1 concentrations, and multi-omics research to uncover the relevant biological mechanisms.

## CONCLUSIONS

This MR study provides genetic evidence that smoking initiation is associated with reduced NBL1 expression, whereas maternal smoking around birth and household smoking exposure showed no causal relationship with NBL1. Importantly, these findings do not support a role for smoking-related NBL1 dysregulation in the development of neuroblastoma. Instead, smoking-induced suppression of NBL1 may have greater relevance to the pathogenesis of adult malignancies.

## Supplementary Material



## Data Availability

The datasets used and/or analyzed during the current study are available from the corresponding author on reasonable request.

## References

[1] Tobacco and nicotine. World Health Organization. June 26, 2026. Accessed August 4, 2026. https://www.who.int/news-room/fact-sheets/detail/tobacco

[2] Gandini S, Botteri E, Iodice S, et al. Tobacco smoking and cancer: a meta-analysis. Int J Cancer. 2008;122(1):155-164. doi:10.1002/ijc.23033 17893872

[3] IARC Working Group on the Evaluation of Carcinogenic Risks to Humans. Tobacco smoke and involuntary smoking. IARC Monogr Eval Carcinog Risks Hum. 2004;83:1-1438 15285078 PMC4781536

[4] National Center for Chronic Disease Prevention and Health Promotion (US) Office on Smoking and Health. The Health Consequences of Smoking-50 Years of Progress: A Report of the Surgeon General. Centers for Disease Control and Prevention (US); 2014. Accessed August 4, 2026. https://www.ncbi.nlm.nih.gov/books/NBK179276/24455788

[5] Liu W, Yu W, Zhang K, Wang M. Associations between maternal smoking around birth and hepatocellular carcinoma: a bidirectional two-sample Mendelian randomization study in East Asian populations. Tob Induc Dis. 2026;24(April):52 doi:10.18332/tid/218848 PMC13123645 42058183

[6] Campbell K, Siegel DA, Umaretiya PJ, et al. A comprehensive analysis of neuroblastoma incidence, survival, and racial and ethnic disparities from 2001 to 2019. Pediatr Blood Cancer. 2024;71(1):e30732. doi:10.1002/pbc.30732 37867409 PMC11018254

[7] Newman EA, Abdessalam S, Aldrink JH, et al. Update on neuroblastoma. J Pediatr Surg. 2019;54(3):383-389. doi:10.1016/j.jpedsurg.2018.09.004 30305231

[8] Martinez-Monleon A, Gaarder J, Djos A, Kogner P, Fransson S. Identification of recurrent 3q13.31 chromosomal rearrangement indicates LSAMP as a tumor suppressor gene in neuroblastoma. Int J Oncol. 2023;62(2):27. doi:10.3892/ijo.2023.5475 36601748 PMC9851131

[9] Ozaki T, Enomoto H, Nakamura Y, et al. The genomic analysis of human DAN gene. DNA Cell Biol. 1997;16(9):1031-1039. doi:10.1089/dna.1997.16.1031 9324305

[10] Nolan K, Kattamuri C, Luedeke DM, et al. Structure of neuroblastoma suppressor of tumorigenicity 1 (NBL1): insights for the functional variability across bone morphogenetic protein (BMP) antagonists. J Biol Chem. 2015;290(8):4759-4771. doi:10.1074/jbc.M114.628412 25561725 PMC4335214

[11] Kumar U, Jahnavi G, Biswas B, Alam B, Varshney S. Molecular effect of tobacco on genetic, epigenetic, and metabolic pathways during cancer progression. Cureus. 2026;18(1):e102757. doi:10.7759/cureus.102757 41782786 PMC12954342

[12] Caron H, Peter M, van Sluis P, et al. Evidence for two tumour suppressor loci on chromosomal bands 1p35-36 involved in neuroblastoma: one probably imprinted, another associated with N-myc amplification. Hum Mol Genet. 1995;4(4):535-539. doi:10.1093/hmg/4.4.535 7633401

[13] Nakamura Y, Ozaki T, Nakagawara A, Sakiyama S. A product of DAN, a novel candidate tumour suppressor gene, is secreted into culture medium and suppresses DNA synthesis. Eur J Cancer. 1997;33(12):1986-1990. doi:10.1016/s0959-8049(97)00333-x 9516839

[14] Li MJ, Liu Z, Wang P, et al. GWASdb v2: an update database for human genetic variants identified by genome-wide association studies. Nucleic Acids Res. 2016;44(D1):D869-D876. doi:10.1093/nar/gkv1317 26615194 PMC4702921

[15] Skrivankova VW, Richmond RC, Woolf BAR, et al. Strengthening the reporting of observational studies in epidemiology using mendelian randomization (STROBE-MR): explanation and elaboration. BMJ. 2021;375:n2233. doi:10.1136/bmj.n2233 34702754 PMC8546498

[16] Elsworth B, Lyon M, Alexander T, et al. The MRC IEU OpenGWAS data infrastructure. bioRxiv. Preprint posted online August 10, 2020. doi:10.1101/2020.08.10.244293

[17] Maes HH, Prom-Wormley E, Eaves LJ, et al. A genetic epidemiological mega analysis of smoking initiation in adolescents. Nicotine Tob Res. 2017;19(4):401-409. doi:10.1093/ntr/ntw294 27807125 PMC5896552

[18] Liu M, Jiang Y, Wedow R, et al. Association studies of up to 1.2 million individuals yield new insights into the genetic etiology of tobacco and alcohol use. Nat Genet. 2019;51(2):237-244. doi:10.1038/s41588-018-0307-5 30643251 PMC6358542

[19] Kobayashi H, Satake E, Murata Y, et al. Neuroblastoma suppressor of tumorigenicity 1 is associated with the severity of interstitial fibrosis and kidney function decline in IgA nephropathy. J Nephrol. 2023;36(8):2245-2256. doi:10.1007/s40620-023-01704-x 37436574

[20] Hemani G, Zheng J, Elsworth B, et al. The MR-Base platform supports systematic causal inference across the human phenome. Elife. 2018;7:e34408. doi:10.7554/eLife.34408 29846171 PMC5976434

[21] Bowden J, Del Greco M F, Minelli C, Davey Smith G, Sheehan N, Thompson J. A framework for the investigation of pleiotropy in two-sample summary data Mendelian randomization. Stat Med. 2017;36(11):1783-1802. doi:10.1002/sim.7221 28114746 PMC5434863

[22] Bowden J, Davey Smith G, Haycock PC, Burgess S. Consistent estimation in Mendelian randomization with some invalid instruments using a weighted median estimator. Genet Epidemiol. 2016;40(4):304-314. doi:10.1002/gepi.21965 27061298 PMC4849733

[23] Hartwig FP, Davey Smith G, Bowden J. Robust inference in summary data Mendelian randomization via the zero modal pleiotropy assumption. Int J Epidemiol. 2017;46(6):1985-1998. doi:10.1093/ije/dyx102 29040600 PMC5837715

[24] Mikshowsky AA, Gianola D, Weigel KA. Assessing genomic prediction accuracy for Holstein sires using bootstrap aggregation sampling and leave-one-out cross validation. J Dairy Sci. 2017;100(1):453-464. doi:10.3168/jds.2016-11496 27889124

[25] Capasso M, Diskin SJ, Totaro F, et al. Replication of GWAS-identified neuroblastoma risk loci strengthens the role of BARD1 and affirms the cumulative effect of genetic variations on disease susceptibility. Carcinogenesis. 2013;34(3):605-611. doi:10.1093/carcin/bgs380 23222812 PMC3716226

[26] Cui C, Zhang H, Guo LN, et al. Inhibitory effect of NBL1 on PDGF-BB-induced human PASMC proliferation through blockade of PDGFβ-p38MAPK pathway. Biosci Rep. 2016;36(4):e00374. doi:10.1042/BSR20160199 27474499 PMC5006314

[27] Strzelak A, Ratajczak A, Adamiec A, Feleszko W. Tobacco smoke induces and alters immune responses in the lung triggering inflammation, allergy, asthma and other lung diseases: a mechanistic review. Int J Environ Res Public Health. 2018;15(5):1033. doi:10.3390/ijerph15051033 29883409 PMC5982072

[28] Ohtori S, Yamamoto T, Ino H, et al. Differential screening-selected gene aberrative in neuroblastoma protein modulates inflammatory pain in the spinal dorsal horn. Neuroscience. 2002;110(3):579-586. doi:10.1016/s0306-4522(01)00590-5 11906795

[29] Chen K, Wei X, Zhang W, Wang R, Wang Y, Yang L. Bone morphogenetic protein 4 derived from the cerebrospinal fluid in patients with postherpetic neuralgia induces allodynia via the crosstalk between microglia and astrocyte. Brain Behav Immun. 2024;119:836-850. doi:10.1016/j.bbi.2024.05.007 38735405

[30] Murillo-Garzón V, Kypta R. WNT signalling in prostate cancer. Nat Rev Urol. 2017;14(11):683-696. doi:10.1038/nrurol.2017.144 28895566

[31] Lu L, Zha Z, Zhang P, et al. Neuron-specific enolase promotes stem cell-like characteristics of small-cell lung cancer by downregulating NBL1 and activating the BMP2/Smad/ID1 pathway. Oncogenesis. 2022;11(1):21. doi:10.1038/s41389-022-00396-5 35487890 PMC9054797

